# Impact of *pentraxin 3* genetic variants on uterine cervical cancer clinicopathologic characteristics

**DOI:** 10.7150/ijms.57886

**Published:** 2021-04-07

**Authors:** Yi-Hung Sun, Ying-Hsiang Chou, Chun-Hao Wang, Yi-Hsuan Hsiao, Chung-Yuan Lee, Shun-Fa Yang, Po-Hui Wang

**Affiliations:** 1 Institute of Medicine, Chung Shan Medical University, Taichung, Taiwan.; 2 Department of Obstetrics and Gynecology, Chi-Mei Foundation Medical Center, Tainan, Taiwan.; 3 Department of Medical Imaging and Radiological Sciences, Chung Shan Medical University, Taichung, Taiwan.; 4 Department of Radiation Oncology, Chung Shan Medical University Hospital, Taichung, Taiwan.; 5 Department of Medicine, National Taiwan University, Taipei, Taiwan.; 6 School of Medicine, Chung Shan Medical University, Taichung, Taiwan.; 7 Department of Obstetrics and Gynecology, Changhua Christian Hospital, Changhua, Taiwan.; 8 Department of Obstetrics and Gynecology, Chiayi Chang Gung Memorial Hospital Chiayi, Taiwan.; 9 Department of Nursing, Chang Gung University of Science and Technology, Chiayi Campus, Chiayi, Taiwan.; 10 Department of Medical Research, Chung Shan Medical University Hospital, Taichung, Taiwan.; 11 Department of Obstetrics and Gynecology, Chung Shan Medical University Hospital, Taichung, Taiwan.

**Keywords:** *pentraxin 3*, genetic variants, uterine cervical cancer, pelvic lymph node metastasis

## Abstract

The aims of this study were to investigate the relationships among *pentraxin 3* (*PTX3*) genetic variants and development and clinicopathological characteristics of uterine cervical cancer, and patient survival in Taiwanese women. The study enrolled 125 patients with invasive cancer and 98 patients with precancerous lesions of uterine cervix, and 325 control women. *PTX3* genetic variants rs2120243, rs3816527, rs2305619 and rs1840680 were selected and their genotypic distributions were determined by real-time polymerase chain reaction. Our results indicated that patients with genotype CC in *PTX3* rs2120243 and genotype GG in rs1840680 had more chance to have adenocarcinoma but not squamous cell carcinoma, as compared to those with CA/AA and those with GA/AA, respectively. No other clinicopatholgical characteristics were associated with *PTX3* genetic variants. In addition, *PTX3* genetic variants were not associated with 5 years survival of cervical cancer patients. In conclusions, *PTX3* genetic variants are not associated with carcinogenesis and clinicopathological variables of uterine cervix and patient survival in Taiwanese women. The only independent predictor for the 5 years survival is pelvic lymph node metastasis.

## Introduction

Pentraxin 3 (PTX3) is the first member of the pentraxin superfamily of multifunctional and multimeric proteins, and originally found as a long-chain pentameric protein of 40-50 kilodaltons 1-3. It could be produced and released by a variety of cells such as neutrophils, macrophages and fibroblasts 4,5. PTX3 is recognized to be an acute-phase reactive protein because its levels in plasma are low under normal conditions, but elevated rapidly during sepsis and other inflammatory situation and infection 6,7. In addition to the regulation in inflammation, PTX3 also exhibits biological reactions in cancer and tissue repair 8.

When the shared sequence of a gene exhibits the difference in the single nucleotides between the members of a species or paired chromosomes in more than 1% of certain population, single-nucleotide polymorphisms (SNPs) will appear 9. SNPs may influence the gene expression by exerting an impact on the promoter area, exon and 3'-untranslated region, and then genetic susceptibility is involved and affects the development of diseases and cancers such as oral cancer and oral submucous fibrosis 9-12. There are 22 SNPs spaning the *PTX3* gene on chromosome 3. Two of them are situated at intronic regions of the gene (rs2305619 at intron 1, and rs1840680 at intron 2), and one (rs3816527 at exon 2) presenting as a non-synonymous variant, which leads to a single amino acid alanine to aspartic acid change. They may be concerned with the susceptibility to various infections and cancer-related infection 13-15.

Uterine cervical cancer is the fourth most common cancer among women globally, accounting for approximately 580,000 new diagnoses in 2018 16. Approximately 90% of deaths from cervical cancer occur in low- and middle-income countries. The worldwide annual incidence of uterine cervical cancer was reported to be 14.0 per 100,000 populations 17. Uterine cervical cancer ranked the eighth most common malignancy in female individuals based on 2014 Annual Cancer Registry Report in Taiwan. Cervical carcinogenesis is a continuous multistep process. Cervical intraepithelial neoplasias (CINs) can remain persistent progression into invasive cancer.^18^ Cervical intraepithelial neoplasia 1 (CIN 1) is called low-grade squamous intraepithelial lesion in cytology (LSIL; also referred to low-grade CIN or low-grade dysplasia in histology), in which mitotic and immature cells occupy only the lower third of the epithelium; whereas CIN 2 and CIN 3 are denominated (collectively referred to as high-grade squamous intraepithelial lesion, HSIL in cytology; also known as high-grade CIN or high-grade dysplasia in histology) when mitotic and immature cells respectively exist in the middle and upper thirds, according to the Bethesda system 19,20. Approximately 90% of CIN 1 was estimated to regress to normal. In contrast, high-grade CIN progress to invasive cancer in considerable rate 21, and is therefore regarded as precancerous lesions.

*Pentraxin 3* single nucleotide polymorphisms (rs2305619 and rs1840680) and plasma levels have been reported to be correlated with hepatocellular carcinoma 22. The implication of four PTX genetic variants (rs1840680, rs2305619, rs3816527, and rs2120243) has also been investigated and revealed that rs3816527 in smokers is associated with the development of late-stage cancer and increased lymph node metastasis 23. So far, there have been no studies linking *PTX3* genetic variants to cervical cancer tin Taiwanese women. We inferred that different *PTX3* SNPs exert different effects on genes expression and their protein, and therefore exhibit different susceptibilities to the development of cervical neoplasias. Then, we conducted this study to explore the relationships between *PTX3* genetic variants and the development of cervical cancer and patient survival.

## Materials and methods

### Subjects

The design of this retrospective study was to enroll 125 patients with invasive cancer and 98 women with precancerous lesions (high-grade CIN) from the Department of Obstetrics and Gynecology affiliated to Chung Shan Medical University Hospital in Taichung, Taiwan, from February 1994 to February 2015. At the same time, 325 women without previous cervical neoplasias who received routine examinations in the outpatient department of the hospital were transferred to the control group. The control women were included as having no cervical neoplasias based on the normal cytologic report from cervical Papanicolaou smear and the report was further verified by normal colposcopic results in this general examination. All subjects are Taiwanese women living in central Taiwan. The marital status and education level were compatible in patients and controls. Colposcopy-directed cervical biopsy was done and pathological report was obtained to diagnose the patients with invasive cervical cancer and precancerous lesions. Patients with invasive cervical cancer and precancerous lesions are regarded as patients with cervical neoplasia. These patients underwent the standard treatment protocols, which has been revised in accordance with guidelines provided by National Comprehensive Cancer Network. The Institutional Review Board of the Affiliated Hospital of Chung Shan Medical University approved this study (CSMUH number: CS18208) with informed consents.

### Extract deoxyribonucleic acid (DNA) from the blood samples of all female individuals and select *PTX3* genetic variants

Laboratory staff used venipuncture techniques to draw blood samples from all female individuals. The samples were collected into Vacutainer tubes mingled with ethylenediaminetetraacetic acid, and immediately stored at 4 °C. DNA was extracted from white blood cells according to previous study 24. The obtained DNA was then dissolved in pH 7.8 TE buffer. After that, it was quantified by the measurement of OD260. The OD260/OD280 ratio was checked and the range of 1.8-2.0 met our criteria and defined as pure to prevent its cross reactivity from the current homologous RNA in the samples. The final products were then stored at -20 °C and used as templates for the polymerase chain reaction (PCR).

Based on the data of the international HapMap project and the previous investigation of Yeh et al., four genetic variants of *PTX3* were selected 22,23. *PTX3* genetic variant rs2120243 is located at transcription factor binding site on chromosome 3. rs3816527 is at exon 2 on chromosome 3 and is a non-synonymous variant in which amino acid is changed from alanine to aspartic acid. rs2305619 and rs1840680 are intron variants on chromosome 3. *PTX3* SNPs rs2120243, rs3816527, rs2305619 and rs1840680 were detected by ABI StepOne Real-Time PCR System (Applied Biosystems, Foster City, CA, USA), and determined with SDS vers. 3.0 software, as our previous study 24.

### Statistical analysis

The genotypic distribution of *PTX3* genetic variants rs2120243 reached Hardy-Weinberg equilibrium in the control women [χ^2^ value: 2.07, *p*=0.356, degree of freedom (d.f.)=2]. The genotypic frequencies of other *PTX3* genetic variants rs3816527, rs2305619 and rs1840680 all satisfied to the equilibrium (χ^2^ value: 0.39, *p*=0.821, d.f.=2; χ^2^ value: 1.00,* p*=0.607, d.f.=2; χ^2^ value: 1.95, *p*=0.377, d.f.=2; respectively).

Analysis of variance (ANOVA) was performed to compare the age distribution of the studied subjects and Bonferroni test for post hoc analysis. Chi-square test was applied to relate genotypic distributions of *PTX3* genetic variants to the incidence of cervical neoplasias. Because the age of patients suffering from cervical precancerous lesions is earlier than that of cervical invasive cancer, age must be adjusted. The *p* values were determined by chi-square test, logistic or multinomial logistic regression models even for age adjustment. The adjusted odds ratios (AORs) with their 95% confidence intervals (CIs) were used to define the associations among genotypic distributions of *PTX3* genetic variants and the incidence of cervical neoplasias (including precancerous lesions and invasive cancer) using the logistic and multinomial logistic regression models after controlling for age. Chi-square test was applied to associate *PTX3* genetic variants with clinicopathological factors. The Kaplan-Meier curve model (univariate analysis over time) was used to determine the significances of *PTX3* genetic variants and clinicopathological parameters for patient survival, which were significantly related to 5 years survival of patients with invasive cervical cancer. The log-rank test was applied to discriminate the differences among them. The influences of *PTX3* genetic variants and these clinicopathological characteristics on 5 years survival of these patients were dertimined using the Cox proportional hazard model for multivariate analysis in relation to survival time. The hazard ratios (HRs) were therefore calculated. The SPSS, version 18.0 and WinPepi Software, version 10.0 were performed for statistical analysis. *P* <0.05 was known as statistically significant difference.

## Results

The age distribution of cervical neoplasm patients was significantly different from that of control women (50.9 ± 13.8 vs. 44.0 ± 10.0, *p*<0.001). The results by the ANOVA using Bonferroni test for post hoc analysis revealed that the age distribution between cervical cancer patients and control women was statistically different (56.2 ± 12.4 vs. 44.0 ± 10.0, *p*<0.001), and the age distribution between patients with cervical cancer and those with precancerous lesion was also different (56.2 ± 12.4 vs. 44.2 ± 12.5, *p*<0.001). However, the age distribution between patients with precancerous and control women was not statistically different (44.2 ± 12.5 vs. 44.9 ± 10.0, *p*= 1.000).

There was no significant difference for the genetic distribution C/C, C/A and A/A in *PTX3* genetic variant rs2120243 (*p*=0.516) between patients with cervical neoplasias and control women. The distributions of other *PTX3* genetic variants, rs3816527, rs2305619 and rs1840680 showed no significant difference between patients with cervical neoplasias and control women (*p*=0.480, 0.956 and 0.320, respectively; Table 1). After controlling for age, there were still no significant difference in *PTX3* genetic variants between these patients and control women (*p*=0.511, 0.749, 0.956 and 0.342, respectively; Table 1).

After patients with cervical neoplasias group was classified into subgroups of patients with invasive cancer and those with precancerous lesions, there was still no significant difference for the genetic distribution C/C, C/A and A/A in *PTX3* genetic variant rs2120243 among invasive cancer, precancerous and control subgroups (*p*=0.746; Table 2). It also showed no significant difference in the genotypic distributions of other *PTX3* genetic variants, rs3816527, rs2305619 and rs1840680 (*p*=0.228, 0.997 and 0.506, respectively; Table 2). The risk of invasive cancer and precancerous lesion of uterine cervix did not rise for these *PTX3* genetic variants (Table 2).

Compared with CA/AA, patients with cervical invasive carcinoma with genotype CC in *PTX3* rs2120243 had a higher chance of developing adenocarcinoma, but no squamous cell carcinoma (OR for adenocarcinoma: 0.21, 95% CI: 0.05-0.78, CA/AA vs CC; Table 3). There were no relationships of *PTX3* rs2120243 with other clinicopathological parameters. Patients with genotype GG in *PTX3* rs1840680 also had a higher chance to have adenocarcinoma as compared to those with GA/AA (OR for adenocarcinoma: 0.23, 95% CI: 0.06-0.88, GA/AA vs GG). There were still no relationships of *PTX3* rs1840680 with other clinicopathological parameters. Moreover, there were no significant relationships of other *PTX3* genetic variants, rs3816527 and rs2305619, with clinicopathological variables.

In univariate analysis, *PTX3* genetic variants rs2120243 and rs1840680 had nothing to do with 5 years survival in cervical cancer patients (HR: 0.62, 95% CI: 0.24-1.59, *p*=0.318 for rs2120243; HR: 0.76, 95% CI: 0.31-1.89, *p*=0.556 for rs1840680; Table 4). In contrast, HRs with poor 5 years survival could be found in cervical patients with more advanced stages (≥ stage II), deep stromal invasion, large tumor diameter, positive parametrium invasion and positive pelvic lymph node metastasis (Table 4).

In multivariate analysis, *PTX3* genetic variants rs2120243 and rs1840680 were also not associated with 5 years survival. However, HR with significantly poor 5 years survival was only found in cervical patients with positive pelvic lymph node metastasis (HR: 9.79, 95% CI: 2.43-37.75, *p*=0.001; Table 5).

## Discussion

It was reported that *PTX3* is related to various cancers such as prostate cancer and lung cancer 25-28. In addition, it has been found that *PTX3* contributes to carcinogenesis and metastasis of human cervical cancer cells and regarded as a possible biomarker for cervical cancer 29. PTX3 has been identified as a signature of 11 proteins for invasive cervical cancer, both individually and multivariate signatures 30. *PTX3* genetic variant rs2120243 is located at transcription factor binding site on chromosome 3 23. Variation at this site can change transcriptional regulation, which may influence the circulating plasma PTX3 levels 31. rs3816527 is at exon 2 on chromosome 3 and is a non-synonymous variant in which amino acid is changed from alanine to aspartic acid. This could potentially have an interference with the N-terminal-mediated binding of PTX3 to its ligands and affect its function 32. rs2305619 and rs1840680 are intron variants on chromosome 3 23. Previous studies have found that *PTX3* SNPs rs2305619 and rs1840680 have functional significance and revealed that the mutant allele A of *PTX3* rs2305619 and rs1840680 is related to higher plasma levels of *PTX3* protein 31,33. PTX genetic variants may have an impact on the expression of *PTX3* gene or its protein levels. The implication of four *PTX3* genetic variants rs1840680, rs2305619, rs3816527, and rs2120243 has been investigated by few authors 22,23. To date, no study investigates the involvement of *PTX3* genetic variants in the development of cervical cancer and patient prognosis in Taiwanese women. Thus, we designed this study to investigate the significance of *PTX3* genetic variants in the occurrence of cervical cancer.

In this study, four *PTX3* gene variants rs2120243, rs3816527, rs2305619 and rs1840680 in Taiwanese women with cervical neoplasias and control women did not find significant differences in genotypic distribution. Even after subdividing patients with cervical neoplasias into invasive and precancerous subgroups and adjusting for age, no significant genotypic distribution of these *PTX3* variants was found. As far as we know, few studies have revealed a significant association between *PTX3* genetic variants and cancer development. However, it was showed that *PTX3* genetic variants rs2305619 and rs3816527 were associated with invasive mold infections in acute leukemia patients undergoing intense chemotherapy 13. Fisher et al. reported that *PTX3* genetic variants rs7252229 and rs3816527 may be associated with invasive aspergillosis following hematopoietic cell transplantation 34. *PTX3* SNP rs3816527 has been reported to be associated with invasive pulmonary aspergillosis in non-neutropenic patients 35. Zhang et al. suggested that *PTX3* genetic variant rs3845978 was related to ankylosing spondylitis, but rs2305619 was not 36. In contrast, Barbati et al. found that *PTX3* polymorphisms, rs2305619, rs3816527, rs1840680, were not linked to the risk of acute myocardial infarction 33.

We further investigated the relationships between *PTX3* genetic variants and clinopathological parameters of cervical cancer. Compared with CA/AA and GA/AA, patients with genotype CC in *PTX3* rs2120243 and genotype GG in rs1840680 were more likely to develop adenocarcinoma. Otherwise, *PTX3* genetic variants had nothing to do with clinicopathological characteristics. However, a high PTX3 expression has been proved to be related to pancreatic cancer metastasis 27. In addition, several studies have demonstrated the role of PTX3 in epithelial cancer metastasis because of epithelial-mesenchymal transition 37-39. It was suggested that oral cancer patients with *PTX3* genetic variant rs3816527 in smoking were linked to the development of late-stage cancer and the increase of lymph node metastasis 23. Carmo et al. demonstrated a significant association between *PTX3* polymorphisms and hepatocellular occurrence 22. *PTX3* rs2305619 polymorphism has been reported to be associated with Child-Pugh scores B and C in hepatocellular carcinoma individuals. Brunel et al. found that the 6-month cumulative incidence of invasive mold infections for patients, carrying two copies of the minor allele of *PTX3* SNPs rs2305619 and/or rs3816527, who had acute myeloid leukemia, acute lymphoblastic leukemia, or refractory anemia with excess blasts-2 and received intense chemotherapy, was significantly different from those without these single SNPs (21% vs 10%; log-rank test *p*=0.04) in the Lausanne University Hospital 13. However, based on National Center for Biotechnology Information dbSNP database, the distribution of *PTX3* genetic variants rs2120243, rs3816527, rs2305619 and rs1840680 in Taiwanese are not similar to other population of the world, the conflicting findings in cancer development and progression may occur.

In the univariate analysis of the effects of *PTX3* genetic variants and clinicopathological characteristics on patient survival, although cervical cancer patients with the genotype CC in rs2120243 and the genotype GG in *PTX3* rs1840680 had a higher chance of developing adenocarcinoma, no *PTX3* variants were found to be predictive of 5 years survival. Hazard ratios with poor 5 years survival could be found in cervical patients with more advanced stages (≥ stage II), deep stromal invasion, large tumor diameter, positive parametrium invasion and positive pelvic lymph node metastasis. Moreover, after controlling the clinicopathological parameters through multivariate analysis, we also found that *PTX3* genetic variants had no significant influence on 5 years survival in cervical cancer patients. Pelvic lymph node metastasis was suggested to be the only independent predictor of 5 years survival in cervical cancer patients through multivariate analysis in Taiwanese women among those significant predictor variables in univariate analysis. This suggestion is reasonable because it has been reported that pelvic lymph node metastasis is the most important prognostic indicator for the survival of cervical cancer patients 40,41.

This study has two main limitations. First, we only studied the population of central Taiwan and did not enroll residents from other areas. Therefore, the recruited sample size was relatively small. Thus, statistical analysis of the impact of *PTX3* genetic variants on patient survival may not be enough to achieve a significant correlation. This may limit the external validity of this investigation. Second, women in the control group were recruited from the outpatient clinic of our hospital for routine examination. Due to their conservative attitude, there is no routine detection for human papillomavirus infection. The possible influence of this factor has not been analyzed.

## Figures and Tables

**Table 1:** Genetic variant distributions of *pentraxin 3* in women with cervical neoplasias and control women in Taiwan

Genetic variants	Controls (n =325)	Cervical neoplasias^a^ (n=223)	ORs (95% CIs)	*p* values	AORs (95% CIs)^b^	Adjusted *p* values^b^
**rs2120243**				0.516		0.511
C/C^c^	123	95	1.00		1.00	
C/A	140	92	0.85 (0.59-1.24)	0.399	0.82 (0.55-1.21)	0.315
A/A	28	25	1.16 (0.63-2.11)	0.637	1.08 (0.57-2.02)	0.819
C/C^c^	123	95	1.00	0.570	1.00	0.435
C/A & A/A	168	117	0.90 (0.63-1.29)		0.86 (0.59-1.25)	
C/C & C/A^c^	263	187	1.00	0.434	1.00	0.562
A/A	28	25	1.26 (0.71-2.22)		1.19 (0.66-2.17)	
**rs3816527**				0.480		0.749
A/A^c^	186	143	1.00		1.00	
A/C	101	67	0.86 (0.59-1.26)	0.444	0.88 (0.59-1.31)	0.526
C/C	11	12	1.42 (0.61-3.31)	0.418	1.16 (0.48-2.82)	0.747
A/A^c^	186	143	1.00	0.640	1.00	0.627
A/C & C/C	112	79	0.92 (0.64-1.32)		0.91 (0.62-1.33)	
A/A & A/C^c^	287	210	1.00	0.347	1.00	0.674
C/C	11	12	1.49 (0.65-3.44)		1.21 (0.50-2.91)	
**rs2305619**				0.956		0.956
G/G^c^	130	95	1.00		1.00	
G/A	149	103	0.95 (0.66-1.36)	0.765	0.95 (0.65-1.39)	0.797
A/A	34	24	0.97 (0.54-1.74)	0.908	0.93 (0.51-1.71)	0.819
G/G^c^	130	95	1.00	0.771	1.00	0.771
G/A & A/A	183	127	0.95 (0.67-1.35)		0.95 (0.66-1.36)	
G/G & G/A^c^	279	198	1.00	0.985	1.00	0.879
A/A	34	24	1.00 (0.57-1.73)		0.96 (0.54-1.70)	
**rs1840680**				0.320		0.342
G/G^c^	124	102	1.00		1.00	
G/A	142	91	0.78 (0.54-1.13)	0.188	0.76 (0.52-1.12)	0.170
A/A	29	26	1.09 (0.60-1.97)	0.775	1.02 (0.55-1.90)	0.949
G/G^c^	124	102	1.00	0.305	1.00	0.254
G/A & A/A	171	117	0.83 (0.59-1.18)		0.81 (0.56-1.17)	
G/G & G/A^c^	266	193	1.00	0.459	1.00	0.602
A/A	29	26	1.24 (0.71-2.17)		1.17 (0.65-2.11)	

Statistical analysis: logistic regression model or chi-square test. ^a^Cervical neoplasias comprised precancerous lesions and invasive cancer of the uterine cervix. ^b^The adjusted p values as well as adjusted odds ratios (AORs) and their 95% confident intervals (95% CIs) were determined through logistic regression model after controlling age. ^c^Used as a reference for comparison to calculate the odds ratios of other genotypes.

**Table 2:** Genetic variant distributions of *pentraxin 3* in women with uterine cervical invasive cancer or precancerous lesion and control women in Taiwan

Genetic variants	Controls (n =325)	Pre-cancerous lesions (n =98)	Invasive cancer (n =125)	*p* values	AORs (95% CIs)^a^	Ad. *p* values	AORs (95% CIs)^b^	Ad. *p* values
**rs2120243**								
C/C^c^	123	39	56	0.746	1.00		1.00	
C/A	140	42	50		0.94 (0.57-1.55)	0.817	0.69 (0.41-1.14)	0.146
A/A	28	12	13		1.34 (0.62-2.88)	0.459	0.87 (0.39-1.94)	0.728
C/C^c^	123	39	56	0.644	1.00		1.00	
C/A & A/A	168	54	63		1.01 (0.63-1.62)	0.972	0.72 (0.44-1.16)	0.176
C/C & C/A^c^	263	81	106	0.660	1.00		1.00	
A/A	28	12	13		1.38 (0.67-2.84)	0.383	1.05 (0.49-2.25)	0.904
**rs3816527**								
A/A^c^	186	59	84	0.228	1.00		1.00	
A/C	101	35	32		1.09 (0.67-1.77)	0.717	0.67 (0.39-1.13)	0.133
C/C	11	3	9		0.85 (0.23-3.15)	0.807	1.36 (0.48-3.80)	0.563
A/A^c^	186	59	84	0.556	1.00		1.00	
A/C & C/C	112	38	41		1.07 (0.67-1.71)	0.781	0.75 (0.46-1.23)	0.249
A/A & A/C^c^	287	94	116	0.245	1.00		1.00	
C/C	11	3	9		0.82 (0.22-3.01)	0.767	1.54 (0.56-4.25)	0.407
**rs2305619**								
G/G^c^	130	41	54	0.997	1.00		1.00	
G/A	149	46	57		0.98 (0.61-1.59)	0.935	0.90 (0.55-1.46)	0.659
A/A	34	11	13		1.02 (0.48-2.20)	0.953	0.86 (0.39-1.88)	0.697
G/G^c^	130	41	54	0.928	1.00		1.00	
G/A & A/A	183	57	70		0.99 (0.62-1.57)	0.960	0.89 (0.56-1.42)	0.616
G/G & G/A^c^	279	87	111	0.984	1.00		1.00	
A/A	34	11	13		1.03 (0.50-2.13)	0.927	0.91 (0.43-1.91)	0.794
**rs1840680**								
G/G^c^	124	41	61	0.506	1.00		1.00	
G/A	142	43	48		0.91 (0.56-1.49)	0.720	0.61 (0.37-1.02)	0.058
A/A	29	12	14		1.24 (0.58-2.65)	0.580	0.84 (0.39-1.84)	0.669
G/G^c^	124	41	61	0.352	1.00		1.00	
G/A & A/A	171	55	62		0.97 (0.61-1.55)	0.896	0.66 (0.41-1.05)	0.081
G/G & G/A^c^	266	84	109	0.734	1.00		1.00	
A/A	29	12	14		1.30 (0.63-2.66)	0.475	1.07 (0.51-2.24)	0.865

^a^Adjusted *p* values and adjusted odds ratios with their 95% CIs were assessed using multinomial logistic regression models after controlling for age between patients with uterine cervical precancerous lesions and control women. ^b^Adjusted *p* values and adjusted odds ratios with their 95% CIs were assessed using multinomial logistic regression models after controlling for age between patients with uterine cervical invasive cancer and control women. ^c^Used as a reference for comparison to calculate the odds ratios of other genotypes. AORs, adjusted odds ratios; 95% CIs, 95% confidence intervals; Ad. *p*, adjusted *p*.

**Table 3:** Associations between genotypic distributions of *pentraxin 3* and clinicopathological characteristics of the patients with cervical invasive cancer

Characteristics^a^	rs2120243				rs1840680			
	CC^b^	CA/AA	ORs (95% CIs)	*p* value	GG^b^	GA/AA	ORs (95% CIs)	*p* value
**Clinical stage**				0.405				0.854
stage I^b^	31	39	1.00		35	36	1.00	
≥ stage II	25	23	0.73 (0.35-1.53)		26	25	0.94 (0.46-1.92)	
**Pathologic type**				**0.012**				**0.021**
squamous cell carcinoma^b^	45	60	1.00		50	59	1.00	
adenocarcinoma	11	3	0.21 (0.05-0.78)		11	3	0.23 (0.06-0.88)	
**Cell grading**				0.433				0.290
well (grade 1)^b^	10	8	1.00		11	7	1.00	
moderate & poor (grades 2/3)	46	55	1.50 (0.55-4.10)		50	55	1.73 (0.62-4.80)	
**Stromal invasion depth**				0.530				0.783
≤ 10 mm^b^	27	33	1.00		31	29	1.00	
> 10 mm	26	25	0.79 (0.37-1.66)		27	28	1.11 (0.53-2.31)	
**Tumor diameter**				0.689				0.935
≤ 4cm**^b^**	31	36	1.00		34	33	1.00	
> 4 cm	25	25	0.86 (0.41-1.79)		27	27	1.03 (0.50-2.11)	
**Parametrium**				0.968				0.526
no invasion^b^	36	39	1.00		40	36	1.00	
invasion	20	22	1.02 (0.48-2.16)		21	24	1.27 (0.61-2.66)	
**Vagina**				0.190				0.207
no invasion^b^	33	43	1.00		36	42	1.00	
invasion	23	18	0.60 (0.28-1.29)		25	18	0.62 (0.29-1.31)	
**Pelvic lymph node**				0.181				0.441
no metastasis^b^	39	49	1.00		43	46	1.00	
metastasis	17	12	0.56 (0.24-1.32)		18	14	0.73 (0.32-1.64)	

Statistical analyses: chi-square; ^a^Some clinicopathological data could not be obtained from the patients with cervical invasive cancer because of incomplete medical charts or records. ^b^As a reference. ORs, odds ratios; 95% CIs, 95% confidence intervals.

**Table 4:** Univariate analysis of *pentraxin 3 (PTX3)* genetic variants rs2120243 and rs1840680 and clinicopathological characteristics for 5 years survival in cervical cancer patients

Variables^a^	5 years survival			
	+	─	*p* value	ORs (95% CIs)
**rs2120243**			0.318	
CC^b^	45	11		1.00
CA/AA	53	7		0.62 (0.24-1.59)
**rs1840680**			0.556	
GG^b^	50	77		1.00
GA/AA	51	8		0.76 (0.31-1.89)
**Clinical stage**			0.016	
stage I^b^	62	6		1.00
≥ stage II	39	14		3.23 (1.24-8.40)
**Pathologic type**			0.253	
squamous cell carcinoma^b^	92	16		1.00
adenocarcinoma	10	4		1.90 (0.63-5.67)
**Cell grading**			0.915	
well (grade 1)^b^	15	3		1.00
moderate & poor (grades 2/3)	87	17		1.07 (0.31-3.65)
**Stromal invasion depth**			0.011	
≤10 mm^b^	53	4		1.00
> 10 mm	42	15		4.18 (1.39-12.61)
**Tumor diameter**			0.010	
≤ 4cm^b^	59	5		1.00
>4 cm	41	15		3.77 (1.37-10.37)
**Parametrium**			0.015	
no invasion^b^	67	7		1.00
invasion	33	13		3.148 (1.25-7.88)
**Vagina**			0.129	
no invasion^b^	66	10		1.00
invasion	34	10		1.96 (0.82-4.75)
**Pelvic lymph node**			<0.001	
no metastasis^b^	82	5		1.00
metastasis	18	15		9.33 (3.39-25.71)

Statistical analyses: Kaplan-Meier curve model; ^a^Some clinicopathological data could not be obtained from the patients with cervical invasive cancer because of incomplete records of medical chart. ^b^As a reference. Survival; +, survival, -, mortality.

**Table 5:** Multivariate analysis for the associations of *pentraxin 3 (PTX3)* genetic variants rs2120243 and rs1840680 as well as various clinicopatholgical characteristics with 5 years survival of cervical cancer patients

Variables	Overall survival	
	*p* value	HR & 95% CI^b^
***PTX3* genetic polymorphisms**		
rs2120243 CA/AA vs. CC^a^	0.952	0.001 (u.a.)
rs1840680 GA/AA vs. GG^a^	0.950	0.002 (u.a.)
**Clinicopathological characteristics**		
**Pelvic lymph node**		
metastasis vs. no metastasis^a^	**0.001**	9.79 (2.43-37.75)

Statistical analyses: Cox proportional hazard model; ^a^As a comparison reference; ^b^HR, hazard ratio and 95% CI, 95% confidence interval for ***PTX3*** genetic variants rs2120243 and rs1840680 and clinicopathological variables, compared to their respective controls. u.a.: unavailable.
